# Ubiquitous Promoter-Localization of Essential Virulence Regulators in *Francisella tularensis*

**DOI:** 10.1371/journal.ppat.1004793

**Published:** 2015-04-01

**Authors:** Kathryn M. Ramsey, Melisa L. Osborne, Irina O. Vvedenskaya, Cathy Su, Bryce E. Nickels, Simon L. Dove

**Affiliations:** 1 Division of Infectious Diseases, Boston Children’s Hospital, Harvard Medical School, Boston, Massachusetts, United States of America; 2 Department of Genetics and Waksman Institute, Rutgers University, Piscataway, New Jersey, United States of America; Emory University School of Medicine, UNITED STATES

## Abstract

*Francisella tularensis* is a Gram-negative bacterium whose ability to replicate within macrophages and cause disease is strictly dependent upon the coordinate activities of three transcription regulators called MglA, SspA, and PigR. MglA and SspA form a complex that associates with RNA polymerase (RNAP), whereas PigR is a putative DNA-binding protein that functions by contacting the MglA-SspA complex. Most transcription activators that bind the DNA are thought to occupy only those promoters whose activities they regulate. Here we show using chromatin immunoprecipitation coupled with high-throughput DNA sequencing (ChIP-Seq) that PigR, MglA, and SspA are found at virtually all promoters in *F*. *tularensis* and not just those of regulated genes. Furthermore, we find that the ability of PigR to associate with promoters is dependent upon the presence of MglA, suggesting that interaction with the RNAP-associated MglA-SspA complex is what directs PigR to promoters in *F*. *tularensis*. Finally, we present evidence that the ability of PigR (and thus MglA and SspA) to positively control the expression of genes is dictated by a specific 7 base pair sequence element that is present in the promoters of regulated genes. The three principal regulators of virulence gene expression in *F*. *tularensis* therefore function in a non-classical manner with PigR interacting with the RNAP-associated MglA-SspA complex at the majority of promoters but only activating transcription from those that contain a specific sequence element. Our findings reveal how transcription factors can exert regulatory effects at a restricted set of promoters despite being associated with most or all. This distinction between occupancy and regulatory effect uncovered by our data may be relevant to the study of RNAP-associated transcription regulators in other pathogenic bacteria.

## Author Summary

Most transcription regulators are found only at those promoters they control. Here we show that the most prominent regulators of virulence gene expression in *Francisella tularensis* are found ubiquitously at promoters including those they do control and those they do not. Furthermore, we present evidence that these regulators—the RNA polymerase-associated SspA family members MglA and SspA, and the putative DNA-binding protein PigR—exert their coordinate regulatory effects only at promoters that contain a small DNA sequence element. Our findings reveal how transcription factors can associate with many promoters but only exert regulatory effects at a few. They also have implications for how SspA family members and other RNAP-associated transcription regulators might exert their effects in other pathogens.

## Introduction

*Francisella tularensis* is a Gram-negative bacterium and the aetiological agent of tularemia, a disease that can be fatal in humans [1]. This pathogen is highly infectious, with as few as 10 organisms constituting an infectious dose, and is a potential bioweapon [2]. The ability of *F*. *tularensis* to cause disease is dependent principally upon its ability to grow within macrophages [1,3–5]. Prominent amongst those genes that are essential for the intramacrophage growth and survival of *F*. *tularensis* are those located on the Francisella pathogenicity island (FPI), which are thought to have been acquired through horizontal transfer [6–8]. Genes on the FPI encode a type VI secretion system that may secrete effector proteins into cells of the host [9,10], thereby enabling the organism to escape the so-called Francisella-containing vacuole and to replicate freely within the macrophage cytosol [4,11,12].

Expression of the genes on the FPI is dependent upon the coordinate activities of three regulators [13–18]. Two of these, MglA and SspA, belong to the stringent starvation protein A (SspA) family of proteins and form a heteromeric complex that associates with RNA polymerase (RNAP) [13,15,19]. The other is a putative DNA-binding protein called PigR (also known as FevR in *F*. *novicida*) that works in concert with MglA and SspA by contacting the RNAP-associated MglA-SspA complex directly [16–18]. MglA, SspA, and PigR also positively control the expression of virulence genes outside the FPI, and are thought to control the expression of ~100 genes in total, including many whose roles in virulence are not yet known [14–17]. The findings that MglA, SspA, and PigR are essential for intramacrophage growth and for virulence underscores the indispensible roles these regulators play in the coordinate control of virulence gene expression in *F*. *tularensis* [16,17,20].

According to the current view for how MglA, SspA, and PigR control the expression of virulence genes, PigR functions like a classical transcription activator, binding specifically to a DNA sequence present at the promoters of regulated genes; thus, contact between DNA-bound PigR and the RNAP-associated MglA-SspA complex would stabilize the binding of RNAP to those promoters that contain a PigR binding site [17,18]. However, it is not known whether the promoters of MglA/SspA/PigR-regulated genes contain a specific sequence element that confers responsiveness to PigR. If PigR were indeed to function like a classical transcription activator it would be expected to be located at only those promoters it regulates, and it would be predicted to bind to DNA recognition sites associated with target promoters regardless of whether or not the MglA-SspA complex were present in the cell. Indeed, most classical transcription activators are thought to bind specific sites on the DNA prior to interacting with RNAP [21,22]. Another prediction from the current model is that PigR interacts with the MglA-SspA complex that is associated with the RNAP holoenzyme (the form of the enzyme that contains the σ factor) during transcription initiation [17,18]. However, it is unknown whether the MglA-SspA complex is associated with the RNAP holoenzyme during transcription initiation or the RNAP core enzyme during transcription elongation, or both.

Using chromatin immunoprecipitation followed by high-throughput DNA sequencing (ChIP-Seq), we show that PigR, MglA, and SspA are present at virtually all detected promoters in *F*. *tularensis*. We also demonstrate that PigR requires MglA (and thus presumably the MglA-SspA complex) in order to specifically associate with promoters. Finally, we present evidence that the promoters of PigR-regulated genes contain a specific sequence motif that is both necessary and sufficient for PigR-mediated control. Our findings reveal that the most prominent regulators of virulence gene expression in *F*. *tularensis* are found at essentially all promoters but only positively control those that contain a specific sequence element.

## Results

### Defining promoter regions in *F*. *tularensis* using ChIP-Seq

**Fig 1:**
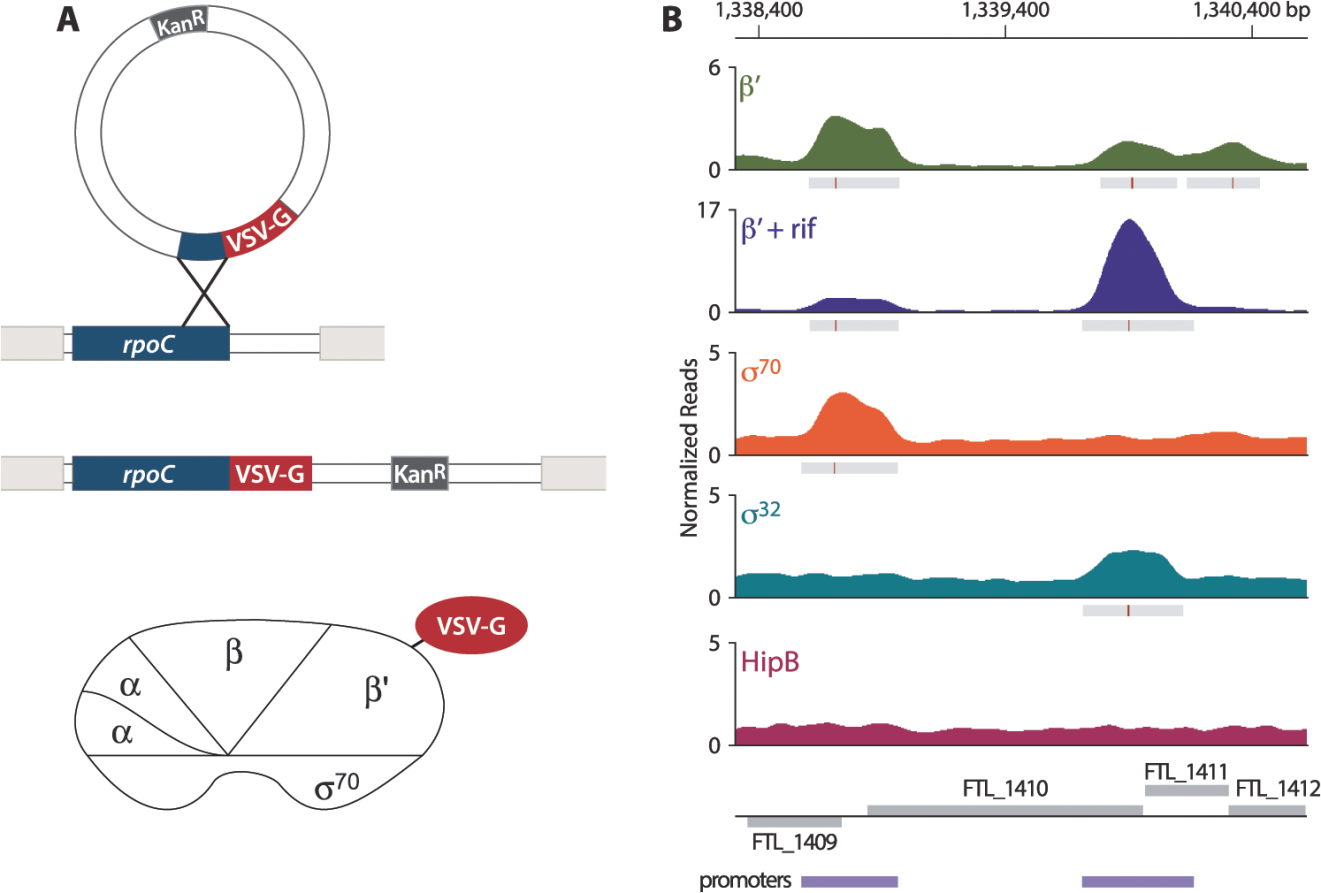
Identification of promoters in *F*. *tularensis* using ChIP-Seq. (A) Schematic representation of the VSV-G tag integration vector and its use to construct the LVS β′-V strain that synthesizes the β′ subunit of RNAP with a VSV-G tag (β′-V) at native levels. (B) A representative illustration of the density of normalized mapped sequencing reads (Y-axis) along a region of the chromosome (X-axis) after ChIP-Seq of each epitope-tagged factor: β′ (green), β′+ rif (purple), σ^70^ (orange), σ^32^ (cyan), and HipB (dark pink). Gray boxes below the read density plot indicate areas of significantly enriched reads; red lines indicate sites of maximum enrichment. Promoter regions, defined as areas with significant enrichment of β′ + rif, σ^32^, or σ^70^ with the chromosome, are indicated by the purple boxes below the gene annotations.

In order to address the question of whether PigR specifically associates with the promoters of regulated genes, we first sought to define the locations of promoters on a genome-wide basis in *F*. *tularensis*. To do this, we determined the locations of the β′ subunit of RNA polymerase (RNAP) on the *F*. *tularensis* chromosome using ChIP-Seq. To immunoprecipitate the β′ subunit of RNAP, we constructed a strain of *F*. *tularensis* LVS in which the chromosomal copy of the *rpoC* gene was modified to encode β′ with a vesicular stomatitis virus-glycoprotein (VSV-G) epitope tag fused to its C-terminus (Fig 1A). This results in cells of LVS which synthesize the β′ subunit of RNAP with a VSV-G tag (β′-V) at native levels. Because β′ is a core subunit of RNAP, β′ is expected to be found at both promoter regions and within actively transcribed genes. Thus, in order to use β′-V to specifically identify the locations of promoters, we performed ChIP-Seq after treatment of the LVS β′-V cells with the RNAP inhibitor rifampicin (rif) to effectively trap RNAP at promoters [23,24] (Fig 1B). By determining the location of the β′ subunit of RNAP in cells treated with rif, we identified 526 promoter regions in *F*. *tularensis* LVS (S1 Table).

*F*. *tularensis* encodes two σ factors: σ^70^, the so-called housekeeping σ factor, and σ^32^, the so-called heat-shock σ factor [25]. As a complementary approach to identify promoters in *F*. *tularensis*, and to determine which promoters are σ^70^-dependent and which are controlled by σ^32^, we performed ChIP-Seq with cells that synthesized epitope-tagged versions of each σ factor. To do this we constructed a strain of LVS that synthesized σ^70^ with a VSV-G epitope tag fused to its C-terminus (LVS σ^70^-V), and another strain of LVS that synthesized σ^32^ with a VSV-G tag fused to its C-terminus (LVS σ^32^-V). As a control we also constructed a strain that synthesized HipB, a predicted site-specific DNA-binding protein [26], with a VSV-G tag fused to its C-terminus (LVS HipB-V). ChIP-Seq with cells of the LVS σ^70^-V strain identified 333 promoter regions, of which 277 (83.2%) overlap with the locations of promoters defined by determining the location of the β′ subunit of RNAP in cells grown in the presence of rif (Fig 1B, S2 Table). ChIP-Seq with cells of the LVS σ^32^-V strain identified only 4 promoter regions (Fig 1B, S3 Table). ChIP-Seq with cells of the LVS HipB-V strain revealed that HipB associates with 26 regions of the chromosome (S4 Table). By defining a promoter as a region with significant enrichment of σ^70^, σ^32^, or the β′ subunit of RNAP in cells grown in the presence of rif, we identified 581 promoter regions in *F*. *tularensis* LVS, 495 (85%) of which were intergenic and 86 (15%) of which were intragenic (S5 Table).

### MglA, SspA, and PigR are found at the majority of promoters in *F*. *tularensis*

Having determined the locations of promoters in *F*. *tularensis* on a genome-wide basis, we next sought to determine at which promoters PigR, MglA, and SspA were located. To do this we utilized a previously constructed strain in which the native chromosomal copy of *mglA* is altered such that it specifies MglA with a TAP (tandem affinity purification) tag fused to its C-terminus [15]. We also constructed two additional strains of LVS: one in which the native chromosomal copy of *pigR* had been altered such that it specified PigR with a VSV-G epitope tag fused to its C-terminus (LVS PigR-V); and another in which the native chromosomal copy of *sspA* had been altered such that it specified SspA with a VSV-G epitope tag fused to its C-terminus (LVS SspA-V).

**Fig 2:**
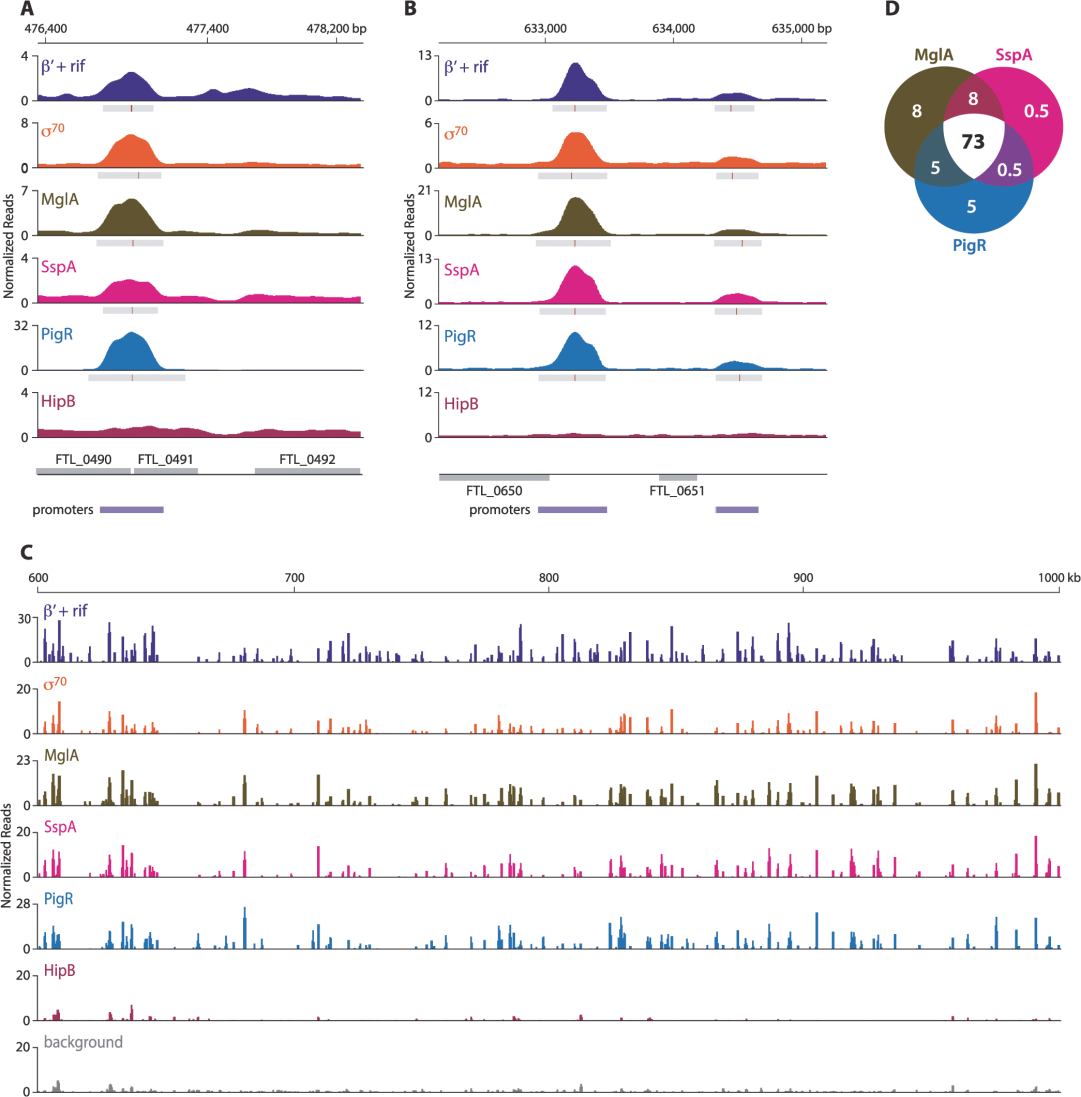
MglA, SspA, and PigR are found ubiquitously at promoter regions. A representative illustration of the density of the normalized mapped sequencing reads after ChIP-Seq of β′+rif (purple), σ^70^ (orange), MglA (brown), SspA (light pink), PigR (blue), and HipB (dark pink) (A) at the FTL_0491 promoter, which is known to be regulated by MglA, SspA, and PigR; (normalized reads are displayed on a linear scale) (B) at the FTL_0650 and FTL_0651 promoters, which are not under the control of MglA, SspA, or PigR (normalized reads are displayed on a linear scale); and (C) across a 400 kb region of the *F*. *tularensis* chromosome (normalized reads are displayed on a log scale). There is significant concordance between the enrichment profiles of β′+rif, σ^70^, MglA, SspA, and PigR. HipB is not specifically enriched at these regions. (D) Venn diagram representing the overlap between MglA, SspA, and PigR peaks at σ^70^-associated promoters. Numbers indicate percent of promoters that are enriched for the indicated transcription factor.

ChIP-Seq with cells of the LVS PigR-V strain, cells of the LVS MglA-TAP strain, and cells of the LVS SspA-V strain revealed that PigR, MglA, and SspA are located at the majority of promoters in *F*. *tularensis* and not just at the promoters of regulated genes (Fig 2, S6 Table). The finding that PigR, MglA, and SspA are found at the promoters of both regulated and non-regulated genes is illustrated in Fig 2A and 2B which show the occupancies of the β′ subunit of RNAP (in the presence of rif), σ^70^, MglA, SspA, PigR, and HipB at the FTL_0491, FTL_0650, and FTL_0651 promoter regions as determined by ChIP-Seq. Specifically, Fig 2A shows that PigR, MglA, and SspA are found at the promoter of the FTL_0491 gene, which is an example of a gene that is positively regulated by MglA, SspA, and PigR [14,16] (see also S6 Table), whereas Fig 2B shows that PigR, MglA, and SspA are found at the promoters of the FTL_0650 and FTL_0651 genes, which are examples of genes that are known not to be positively regulated by MglA, SspA, and PigR [14–17]. HipB was not detected at any of these promoters by ChIP-Seq (Fig 2A and 2B) indicating the specificity of the observed associations of PigR, MglA, and SspA with these promoters. In contrast, HipB is specifically enriched upstream of the *hipB* gene (S1 Fig), suggesting that in *F*. *tularensis* HipB may control its own expression.

The finding that PigR, MglA, and SspA are found at the majority of promoters in *F*. *tularensis* is illustrated in Fig 2C which shows the locations and degrees of occupancy of the β′ subunit of RNAP (in cells grown in the presence of rif), σ^70^, MglA, SspA, PigR, and HipB over a representative 400 kb region of the *F*. *tularensis* chromosome. Comparison between the regions enriched for σ^70^, MglA, SspA, and PigR, together with the relative degree of enrichment, revealed a striking correspondence between the four (Fig 2C). Note that the degree of occupancy of the β′ subunit of RNAP at a particular promoter can differ in the presence and absence of rifampicin (Fig 1B) [27], which may explain why the ChIP-Seq enrichment profile for β′ in cells grown in the presence of rif differs slightly from that of σ^70^ in certain locations (Fig 2C). The concordance among the localization of σ^70^, MglA, SspA, and PigR across the entire *F*. *tularensis* chromosome is demonstrated in Fig 2D which represents the 98% of promoter regions identified by ChIP-Seq of σ^70^ at which at least one of the three factors, MglA, SspA, or PigR, is found; the Venn diagram shows that MglA, SspA, and PigR are found at the majority of promoters identified by detection of σ^70^ (Fig 2D). The identification of PigR at the majority of promoters suggests that PigR is not a regulator that is only found at the promoters of specific target genes.

ChIP-Seq with cells of the LVS PigR-V strain, cells of the LVS MglA-TAP strain, and cells of the LVS SspA-V strain also revealed that PigR, MglA, and SspA are present at promoters together with σ^70^ and are not detected in transcribed regions. This is in contrast to the situation with the β′ subunit of RNAP, which is found both at promoters and within transcribed regions in cells grown in the absence of rif (Fig 1B). These findings suggest that PigR, MglA, and SspA might not be components of the transcription elongation complex and that PigR, MglA, and SspA likely exert their regulatory effects at the level of transcription initiation.

### PigR requires MglA in order to specifically associate with promoters

**Fig 3:**
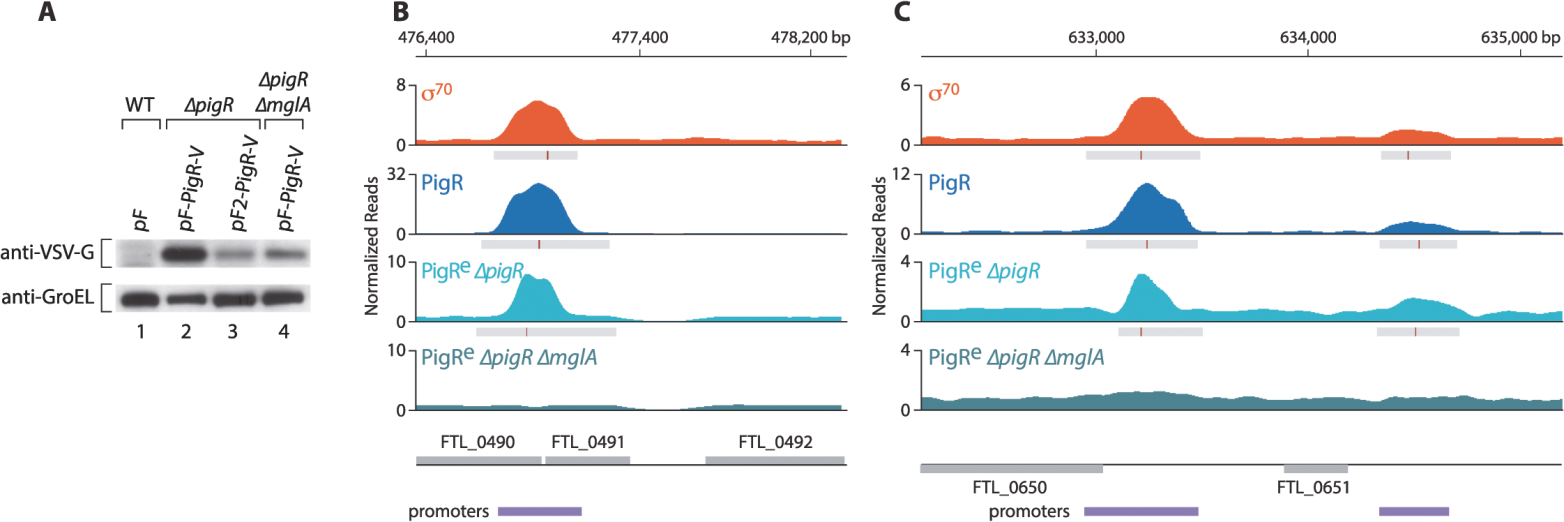
PigR requires MglA to specifically associate with promoter regions. (A) Abundance of ectopically expressed PigR-V as analyzed by Western blot. (*Upper*) Western blot probed with antibody against the VSV-G tag. (*Lower*) Western blot probed with antibody against GroEL serves as a loading control. Wild-type LVS cells containing the empty control vector pF (lane 1); LVS ∆*pigR* mutant cells containing either pF-PigR-V (lane 2) or pF2-PigR-V (lane 3); LVS ∆*pigR* ∆*mglA* mutant cells containing pF-PigR-V (lane 4). (B) and (C) Representative datasets illustrating the density of the normalized mapped sequencing reads after ChIP-Seq with cells of the LVS σ^70^-V strain (orange), cells of the LVS PigR-V strain (blue), cells of the LVS ∆*pigR* mutant strain that ectopically synthesize PigR-V from plasmid pF2-PigR-V (PigR^e^ ∆*pigR*, light blue), and cells of the LVS ∆*pigR* ∆*mglA* mutant strain that ectopically synthesize PigR-V from plasmid pF-PigR-V (PigR^e^ ∆*pigR* ∆*mglA*, blue-green). (B) Ectopically produced PigR-V occupies the promoter of the PigR/MglA/SspA-regulated FTL_0491 gene in cells containing MglA, but not in cells lacking MglA. (C) Ectopically produced PigR-V occupies the promoters of the FTL_0650 and FTL_0651 genes, which are not under the control of PigR/MglA/SspA, only in cells that contain MglA.

Interaction between PigR and the RNAP-associated MglA-SspA complex is required in order for PigR to function coordinately with MglA and SspA [18]. We therefore next asked whether PigR requires the MglA-SspA complex in order to associate with promoter regions in *F*. *tularensis*. Because the expression of *pigR* is dependent upon the presence of MglA [16,17], in order to address this question we performed ChIP-Seq with cells of a ∆*pigR* mutant strain and cells of a *∆pigR ∆mglA* mutant strain that ectopically synthesized similar amounts of plasmid-encoded PigR-V. We found that when supplied from plasmid pF under the control of the strong heterologous *groES* promoter, PigR-V was significantly less abundant in cells of the *∆pigR ∆mglA* mutant strain than in cells of the ∆*pigR* mutant strain (Fig 3A). The *groES* promoter is not positively controlled by MglA, so it is possible that the PigR-V protein is less abundant in cells of a ∆*mglA* mutant strain because it is less stable in the absence of the MglA-SspA complex. Therefore, to be able to compare cells containing similar amounts of PigR, we used the strong *groES* promoter on plasmid pF to drive the synthesis of PigR-V in cells of the *∆pigR ∆mglA* mutant strain and a weakened *groES* promoter lacking an UP-element on plasmid pF2 to drive the synthesis of PigR-V in cells of the ∆*pigR* mutant strain [17] (Figs 3A and S2).

Comparison of the ChIP-Seq results obtained with ectopically produced PigR with those obtained with native PigR revealed that the ectopic synthesis of PigR does not significantly alter the genome-wide locations of this protein. This is illustrated at the promoter for the PigR/MglA/SspA-regulated FTL_0491 gene (Fig 3B), and illustrated at the promoters for the FTL_0650 and FTL_0651 genes, which are not PigR/MglA/SspA-regulated (Fig 3C). Comparison of the ChIP-Seq results obtained with ectopically produced PigR in the presence and absence of MglA revealed a striking difference; we found no specific enrichment of PigR at any promoter in the absence of MglA, or at any other region of the chromosome. This is illustrated at the FTL_0491, FTL_0650 and FTL_0651 promoters in Fig 3B and 3C. These findings indicate that MglA, and by inference the MglA-SspA complex, is required for PigR to specifically associate with promoter regions in *F*. *tularensis*.

### A specific sequence motif is found at the promoters of PigR/MglA/SspA-regulated genes

Although PigR (together with MglA and SspA) is present at the majority of promoters, it appears to only positively regulate the expression of a fraction of the corresponding genes. We therefore reasoned that PigR might function as an activator at specific promoters through recognition of a specific sequence element.

**Fig 4:**
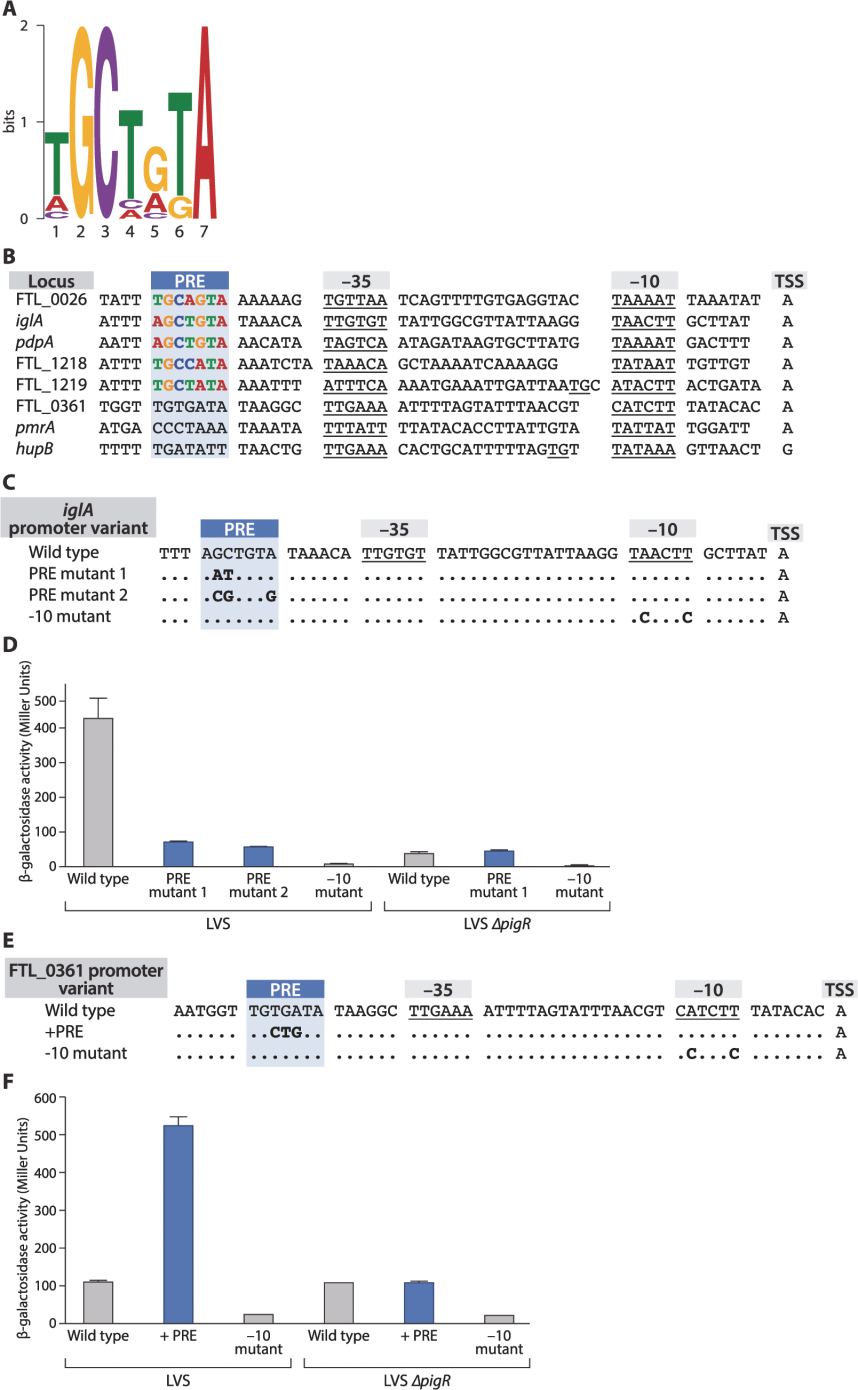
The PigR response element (PRE) is necessary and sufficient for promoters to be controlled by PigR. (A) A logo of the 7 bp consensus PRE sequence motif generated by MEME. (B) Alignment of promoters with mapped transcription start sites and predicted -10 and -35 elements (underlined), including the promoters of five PigR-regulated genes (FTL_0026, *iglA pdpA*, FTL_1218, FTL_1219, FTL_0361) and the promoters of three non-PigR regulated genes (FTL_0361, *pmrA*, *hupB*). The conserved PRE is found 6–7 bp upstream of the -35 element only in those promoters known to be controlled by PigR. (C) Alignment of *iglA* promoter variants. Nucleotide substitutions (in bold) were introduced in the *iglA* promoter fused to the *lacZ* reporter gene and integrated into the FTL_0111 locus. (D) Quantification of *lacZ* expression in strains LVS and LVS ∆*pigR* containing the indicated promoter variants (indicated along the X-axis) by β-galactosidase assay, as measured in Miller Units (Y-axis). (E) Alignment of FTL_0361 promoter variants. Nucleotide substitutions (in bold) were introduced into the FTL_0361 promoter fused to the *lacZ* reporter gene and integrated into the FTL_0361 locus. (F) Quantification of *lacZ* expression in strains LVS and LVS ∆*pigR* containing the indicated promoter variants (indicated along the X-axis) by β-galactosidase assay, as measured in Miller Units (Y-axis). Error bars for the -10 mutant in the LVS strain, the wild type FTL_0361 promoter and the -10 mutant in the LVS ∆*pigR* strain are too small to be illustrated.

To search for a conserved sequence motif in the promoters of genes that are regulated by PigR we first tested whether certain genes previously shown to be regulated by MglA and SspA were also regulated by PigR in *F*. *tularensis*. To do this we quantified specific candidate transcripts in both wild-type LVS cells and in cells of a LVS ∆*pigR* mutant strain using Nanostring (see Materials and Methods; S7 Table). Using MEME [28], we then searched for a specific motif in the promoter regions of genes that (i) were either previously shown to be positively regulated by PigR in LVS by DNA microarray [17], or shown to be positively regulated by PigR in our Nanostring assays (S7 Table), or both, and (ii) contained a region of PigR enrichment upstream from the translation start site, as determined by our ChIP-Seq studies with cells of our LVS PigR-V strain. Eleven genes fit these criteria and a 7 bp motif was found to be present in all 11 of the putative promoter regions analyzed. A logo representing this 7 bp motif, which we have named the PigR response element (PRE) is depicted in Fig 4A.

We next asked whether the PRE was found at a specific location relative to the transcription start site of a regulated promoter. To do this we first determined transcription start sites on a genome-wide basis using RNA-Seq [29,30]. This gave us 453 candidate transcription start sites (see Materials and Methods). This list was parsed further to include only those start sites found within 1 kb upstream of the translational start site of an annotated ORF, and remove from consideration those start sites associated with rRNAs and tRNAs, and those found within repeated sequences annotated as encoding transposases. To obtain a list of transcription start sites that could be independently verified as originating from a detectable promoter we further parsed this list of 197 start sites to include only those found within a region of enrichment for σ^70^, σ^32^, or the β′ subunit of RNAP (in cells grown in the presence of rif) as determined by ChIP-Seq. This gave us transcription start sites with high confidence for 110 promoters, including 3 of the 11 putative promoter regions used to initially identify the PRE through MEME (S8 Table). We then used primer extension to determine transcription start sites for 2 additional promoters of PigR-regulated genes used in the MEME analysis. Through determining the transcription start sites for the promoters driving the expression of 5 independent PigR-regulated genes we were able to infer the sequences and locations of putative -10 and -35 elements for each of these promoters and found that the PRE was either 6 or 7 bp upstream from the predicted -35 element in each case (Fig 4B). Analysis of the 110 promoters from our high quality data set revealed that only 3 of these contained a PRE (see Materials and Methods) and are known to be PigR-regulated, whereas 107 do not contain a PRE and are not known to be PigR-regulated [17] (Fig 4B, S7 and S8 Tables). This suggests that the presence and location (6 or 7 bp upstream of the putative -35 element) of the PRE is specific to PigR-regulated promoters, raising the possibility that PigR may bind directly to this site to activate transcription from those promoters that contain it.

### The PRE is both necessary and sufficient to confer responsiveness to PigR

Having identified a specific conserved sequence element in the same location in the promoters of PigR-regulated genes we sought to determine whether this sequence rendered a particular promoter responsive to PigR. To do this we first constructed a reporter strain of LVS in which one of the two copies of the PigR/MglA/SspA-regulated *iglA* promoter is transcriptionally fused to *lacZ* using a chromosomal integration vector [31]. We also made three additional reporter strains of LVS. Two of these contained different mutations at conserved base pairs in the PRE of the *iglA* promoter-*lacZ* fusion (Fig 4C), whereas the third reporter strain contained mutations in the predicted -10 element of the *iglA* promoter-*lacZ* fusion that would be predicted to abolish promoter activity (Fig 4C) [32]. Finally, we made an additional three reporter strains in cells of the LVS ∆*pigR* mutant strain that contained the wild-type version, a PRE mutant version, or the -10 mutant version of the *iglA* promoter-*lacZ* fusion.

The results depicted in Fig 4D show that mutations in the PRE of the *iglA* promoter reduce expression of the linked *lacZ* reporter gene only when PigR is present (i.e. in cells of LVS but not in cells of the LVS ∆*pigR* mutant strain). Consistent with the idea that these differences are due to a decrease in the activity of the *iglA* promoter, cells of the reporter strains containing mutations in the -10 element that are predicted to decrease the activity of the promoter exhibit dramatically reduced *lacZ* expression (Fig 4D). Note that there are two identical copies of the *iglA* gene in LVS because there are two copies of the FPI in this organism. Only reporter strains carrying wild-type and mutant versions of the *iglA* promoter-*lacZ* fusion integrated at the FTL_0111 locus were used in these experiments, ruling out the possibility that any of the observed differences in *lacZ* expression were due to differences in the location of the reporter in the different strains. Taken together, these findings suggest that residues within the PRE of the *iglA* promoter are important in order for PigR to exert a positive effect on expression of the *iglA* gene.

Having established that conserved base pairs within the PRE are important for expression of a PigR-regulated gene we next asked whether the PRE was sufficient to confer control on a promoter that did not ordinarily contain a PRE. To do this we introduced 3 mutations into the FTL_0361 promoter that generated a consensus PRE 6 bp upstream of the putative -35 element (Fig 4E). We then made reporter strains of LVS and the LVS ∆*pigR* mutant strain that contained the wild-type version, a PRE-containing version, or a -10 mutant version of a FTL_0361 promoter-*lacZ* fusion. The results depicted in Fig 4F show that addition of a PRE to the FTL_0361 promoter results in an increase in expression of the FTL_0361 promoter-*lacZ* fusion only in the presence of PigR (i.e. in cells of the LVS wild-type strain but not in cells of the LVS ∆*pigR* mutant strain). These findings demonstrate that the PRE is sufficient to confer on a promoter the ability to respond to PigR, and by inference, the ability to respond to MglA and SspA.

## Discussion

Using ChIP-Seq we have found that PigR, MglA, and SspA are found at the majority of promoters in *F*. *tularensis*. We have also found that PigR requires the MglA-SspA complex in order to specifically localize to promoter regions. We infer from this that interaction between PigR and the RNAP-associated MglA-SspA complex directs PigR specifically to promoter regions. Despite their ubiquitous presence at promoters, PigR, MglA, and SspA coordinately control the expression of approximately 5% of known genes and we have uncovered the molecular basis for this specificity. In particular, we have identified a 7 bp sequence element that we have called the PRE (the PigR response element), located approximately 6 bps upstream of the putative -35 element of promoters that are positively regulated by PigR/MglA/SspA. The PRE is both necessary and sufficient to confer control by PigR. Our findings indicate that although PigR, MglA, and SspA are present at essentially all promoters, they control the activities of only those promoters that contain a specific sequence element.

Finding PigR, MglA, and SspA at promoters but not within transcribed regions suggests that these proteins are associated with the RNAP holoenzyme and likely exert their regulatory effects at the level of transcription initiation. Consistent with the idea that the MglA-SspA complex interacts with the σ^70^-containing RNAP holoenzyme, σ^70^ together with the core subunits of RNAP and SspA were found to co-purify with MglA in LVS in stoichiometric amounts [15].

**Fig 5:**
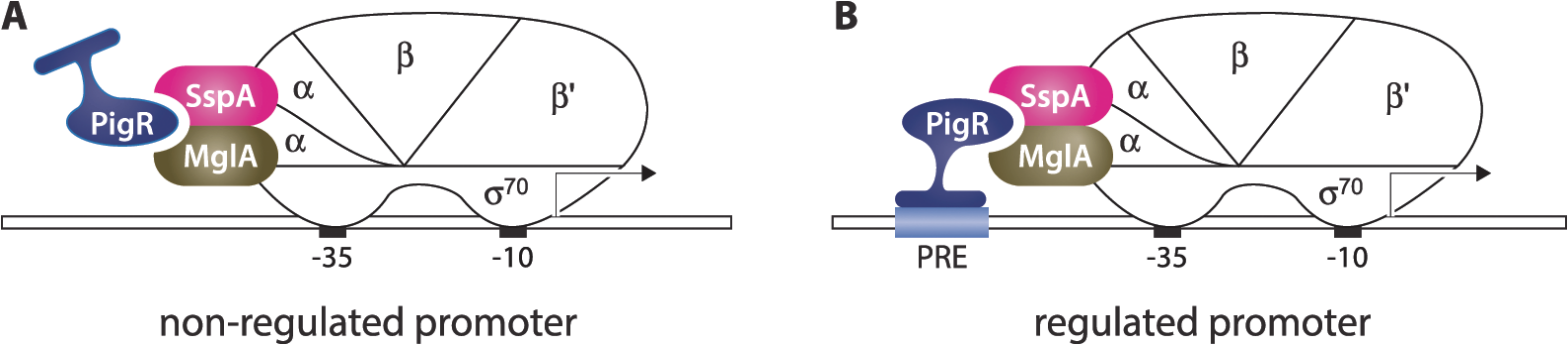
Model for how PigR functions coordinately with the MglA-SspA complex to positively control the expression of genes. PigR associates with RNAP through interaction with the RNAP-associated MglA-SspA complex and is consequently found associated with RNAP at the promoters of both non-regulated (A) and regulated (B) genes. (B) PigR is a DNA-binding protein that binds the PRE present within the promoters of regulated genes; contact between RNAP-bound PigR and the DNA stabilizes the binding of RNAP to the promoter, thereby activating transcription specifically from promoters that contain a PRE. Although for convenience, MglA and SspA are depicted here as interacting with the α subunit of RNAP, it is not known which subunit(s) of RNAP are contacted by the MglA-SspA complex.

PigR contains a putative helix-turn-helix motif, suggesting it might exert its regulatory effects through interaction with the DNA [16,17]. Based on our findings that PigR, MglA, and SspA are present at the majority of σ^70^-dependent promoters in *F*. *tularensis*, together with our identification of the PRE, we propose a model for how PigR works in concert with the MglA-SspA complex to positively regulate the expression of a specific set of genes, including many that are required for virulence (Fig 5). According to this model, PigR is a transcription activator that associates with all promoters through its interaction with the RNAP-associated MglA-SspA complex. However, only at those promoters that contain a PRE does PigR make sufficiently strong contact with the DNA to further stabilize the binding of RNAP and activate transcription. In essence, the model specifies that PigR is an RNAP-associated transcription activator that functions by providing RNAP with an additional DNA-binding domain, conferring on RNAP the ability to form especially stable complexes at promoters that contain a PRE. Note that in this model, PigR/MglA/SspA-regulated promoters are depicted as being recognized by RNAP holoenzyme containing σ^70^ (i.e. are σ^70^-dependent promoters), since our ChIP-Seq studies reveal PigR, MglA, SspA, and σ^70^ are present at many of the same promoter regions. Note also that our model explains only how PigR, together with the MglA-SspA complex, exerts positive effects on gene expression; the small number of genes that are negatively regulated by PigR/MglA/SspA [15–17], may be controlled directly or indirectly by these factors. It is possible that some genes are regulated by PigR/MglA/SspA because they are subject to control by another regulator that is in turn regulated by PigR/MglA/SspA. However, to the best of our knowledge, *pigR* is the only gene encoding a putative DNA-binding protein that is positively regulated by PigR/MglA/SspA [15–17].

Our model specifies that PigR is a DNA-binding protein that associates with RNAP via the MglA-SspA complex prior to promoter binding, and is therefore associated with all promoters, as is supported by our ChIP-Seq data. According to the classical view, transcription activators that bind the DNA and contact RNAP are found only at the promoters of regulated genes and function by first recognizing their respective target sites on the DNA and then, once tethered to the DNA, by contacting RNAP [21,22]. However, there is at least one precedent in the literature for a regulator that appears to manifest ubiquitous promoter localization, while exerting effects at only a subset of promoters. In particular, CarD is an essential RNAP-associated transcription regulator in *Mycobacterium tuberculosis* that has been found to associate with the majority of promoters [33,34]. Although the ability of CarD to bind the DNA is necessary in order for this regulator to stimulate transcription initiation, it is not yet known whether CarD exerts its regulatory effects at promoters through recognition of a specific sequence element [34]. In addition, in *E*. *coli*, members of the MarA family of transcription activators are thought to associate with RNAP prior to DNA-binding and to contact their DNA target sites as a pre-assembled activator-RNAP complex [35–37]. However, to the best of our knowledge, genome-wide location analyses have not been performed on members of the MarA family, and so it is not yet known whether these regulators are found at the majority of promoters in *E*. *coli*.

We note that although PigR is predicted to be a DNA-binding protein, PigR has yet to be shown to be capable of binding the DNA, and need not necessarily exert its regulatory effects through direct interaction with the PRE. It is formally possible that interaction between PigR and the RNAP-associated MglA-SspA complex may enable some other portion of RNAP, such as the C-terminal domain of one of the α subunits [15,38], or perhaps the MglA-SspA complex itself, to interact productively with the PRE, resulting in transcription activation. However, in relation to the latter possibility, SspA family members do not contain any obvious DNA-binding determinants and have not been shown to bind the DNA directly [39,40]. It is important to note that although we found PigR does not specifically associate with the DNA in the absence of MglA, this does not mean that PigR is not a site-specific DNA-binding protein. PigR may need to interact with the MglA-SspA complex in order to specifically interact with the PRE, either because the protein-protein interaction promotes a structural change within PigR that is essential for DNA-binding, or because interaction between PigR and the DNA is too weak to be able to occur in the absence of stabilizing interactions provided by promoter-bound RNAP. Indeed, most sequence-specific transcription regulators bind as dimers to their cognate recognition sites, which are typically 20 bp in length. If PigR does bind the 7 bp PRE directly, this would be more reminiscent of a monomer of a dimeric regulator binding a half-site. Regardless of whether or not the ability of PigR to interact directly with the PRE is essential in order for PigR to exert its regulatory effects, our findings clearly establish the PRE as the sequence element that renders a promoter subject to control by PigR, and thus presumably MglA and SspA as well.

The virulence genes present on the horizontally acquired FPI are the ones that are most strongly regulated by PigR, MglA, and SspA, and it is tempting to speculate that the limited size of the PRE (at 7 bp) may have facilitated the expansion of the PigR/MglA/SspA regulatory network to include these. Only three mutations were required in order to generate a consensus PRE within the FTL_0361 promoter (Fig 4E). More than three mutations would likely have been required had the PRE been closer to 20 as opposed to 7 bp. The relatively short length of the PRE means that relatively few changes would be required to place a particular promoter under the control of PigR/MglA/SspA, including any promoter that might have been acquired from a foreign source through horizontal transfer.

Our ChIP-Seq studies suggest that PigR interacts with the MglA-SspA complex at the majority of promoters. This raises the possibility that through interaction with the MglA-SspA complex, PigR may modulate the activity of any other regulator that functions through interaction with the RNAP-bound MglA-SspA complex. Indeed, it has been suggested that PmrA, another important regulator of virulence gene expression in Francisella, might function through interaction with MglA and SspA [41]. It is therefore conceivable that at some promoters PigR may modulate the activity of PmrA, or vice versa, through competition for a binding surface on the MglA-SspA complex.

The role of the MglA-SspA complex in positively controlling the expression of virulence genes appears to be to simply serve as a contact site on RNAP for PigR. Evidence for other SspA family members serving as contact sites for transcription activators comes from studies of bacteriophage P1 late gene expression; in *E*. *coli*, SspA evidently functions as a co-activator of P1 late gene expression by making simultaneous contact with RNAP and the phage-encoded sequence-specific DNA-binding protein Lpa [39]. However, in the case of Lpa, it is not known whether this regulator is associated with the majority of promoters in *E*. *coli* or just those driving expression of the P1 late genes. SspA family members have been shown to be important for the virulence of a variety of pathogens [42–47]. Serving as a contact site on RNAP for a transcription activator may represent a common mechanism by which SspA family members control the expression of virulence genes in numerous pathogens.

## Materials and Methods

### Growth conditions

*F*. *tularensis* subsp. *holarctica* LVS and its derivatives were grown at 37°C in either Mueller Hinton (MH) broth (Difco), supplemented with glucose (0.1%), ferric pyrophosphate (0.025%), and Isovitalex (2%), or on cysteine heart agar (Difco) medium supplemented with 1% hemoglobin solution (VWR); when appropriate, kanamycin was used for selection at either 5 μg/ml or 10 μg/ml. *Escherichia coli* strain XL1-blue (Stratagene) was used for plasmid construction and, when appropriate, kanamycin was used to select for resistance at 50 μg/ml. *E*. *coli* containing plasmid pBSK *iglA*-*lacZ*, or its derivatives, were grown at 30°C.

### VSV-G tagging integration vectors

A modified version of pEX18Kan (provided by Shite Sebastian and Simon Dillon, Harvard Medical School, Boston, Massachusetts, United States) was used as the vector for VSV-G tagging integration constructs. We have used pEX18Kan for deletion constructs [15,17]; it utilizes a ColE1 origin of replication, which is nonfunctional in LVS, and contains the Tn*903* kanamycin resistance gene (Epicentre) driven by the LVS *groES* promoter. The plasmid pKL01 was generated by first amplifying the last 400 base pairs (bp) of the FTL_1743 locus (*rpoC*), minus the stop codon, by PCR. The 5′ primer contained DNA specifying a KpnI site upstream of the gene fragment. The 3’ primer included DNA containing a NotI site and one extra base pair, encoding a 3 amino acid alanine linker. The linker is followed by DNA specifying the 11 amino acid vesicular stomatitis virus-glycoprotein (VSV-G) epitope tag, followed by a stop codon and DNA specifying an EcoRI site. The corresponding PCR product was digested with KpnI and EcoRI and cloned into pEX18Kan that had been digested with KpnI and EcoRI, generating pKL01. We largely removed the *sacB* gene by digesting with MscI and EcoRV and re-ligating the vector together, resulting in pKL02.

VSV-G tagging integration constructs for *rpoD*, *rpoH*, *sspA*, *pigR*, and *hipB* were generated by amplifying the last 250–400 bp (depending on gene size) of the gene using a 5′ primer containing a KpnI site and a 3′ primer containing a NotI site, which allows each fragment to be fused with DNA specifying the alanine linker and VSV-G epitope tag. Fragments were subcloned into pKL02 that had been digested with KpnI and NotI. Plasmid pKL05 contains the DNA specifying the 3’ end of *rpoD* and was used to generate strain LVS σ^70^-V. Plasmid pKL04 contains the DNA specifying the 3’ end of *rpoH* and was used to generate strain LVS σ^32^-V. Plasmid pKL08 contains the DNA specifying the 3’ end of *pigR* and was used to generate strain LVS PigR-V. Plasmid pCS05 contains the DNA specifying the 3’ end of *hipB* and was used to generate strain LVS HipB-V. Plasmid pKL07 contains the DNA specifying the 3’ end of *sspA*. Because expression of the putative *sspA* operon could potentially be interrupted by plasmid integration, pKL07 was modified to contain an outward facing promoter after plasmid integration. To do this, another *groES* promoter was amplified from LVS genomic DNA and cloned upstream of the *sspA* gene fragment, into the BamHI and PstI sites, resulting in plasmid pKL13, which was used to generate strain LVS SspA-V.

### Plasmids for *Francisella* β-galactosidase reporter assays

The pBSK *iglA*-*lacZ* plasmid (provided by Thomas Kawula, University of North Carolina at Chapel Hill, Chapel Hill, North Carolina, United States) utilizes a ColE1 origin of replication, which is nonfunctional in LVS, contains a kanamycin resistance determinate (*aphA1*), and contains a transcriptional fusion between the *iglA* promoter and the *lacZ* gene [31]. The pBSK *iglA*-*lacZ* plasmid contained two tandem PacI sites, so pMO1, which contains DNA specifying the wild-type *iglA* promoter with a single PacI site before the *lacZ* gene, was generated by digesting pBSK *iglA*-*lacZ* with PacI and NotI and recloning the wild-type *iglA* promoter fragment into the plasmid backbone. The pMO1 plasmid was used to generate LVS P_*iglA*_-lacZ and LVS ∆*pigR* P_*iglA*_-lacZ. Mutations in the PRE of the *iglA* promoter were generated using splicing by overlap extension PCR [48]. The corresponding PCR products were digested with PacI and NotI and cloned into pBSK *iglA*-*lacZ* that had been digested with PacI and NotI to replace the wild-type *iglA* promoter. The pMO2 plasmid contains DNA specifying the *iglA* promoter containing the PRE mutant 1 and was used to generate LVS P_*iglA*_-M1-lacZ and LVS ∆*pigR* P_*iglA*_-M1-lacZ. The pMO3 plasmid contains DNA specifying the *iglA* promoter containing the PRE mutant 2 and was used to generate LVS P_*iglA*_-M2-lacZ. The pMO4 plasmid contains DNA specifying the *iglA* promoter containing the -10 mutant and was used to generate LVS P_*iglA*_-10M-lacZ and LVS ∆*pigR* P_*iglA*_-10M-lacZ.

The promoter region of FTL_0361 was amplified from *F*. *tularensis* LVS genomic DNA using a 5′ primer containing DNA specifying a NotI site upstream of the promoter and the 3’ primer including DNA containing a PacI site downstream of the promoter. The resulting PCR product was digested with PacI and NotI and cloned into pBSK *iglA*-*lacZ* that had been digested with PacI and NotI to replace the *iglA* promoter, generating pMO5. The pMO5 plasmid was used to generate LVS P_*FTL_0361*_-lacZ and LVS ∆*pigR* P_*FTL_0361*_-lacZ. Plasmids containing mutations in the FTL_0361 promoter were generated in the same manner as plasmids containing mutations in the *iglA* promoter. The pMO6 plasmid contains DNA specifying the FTL_0361 promoter containing the PRE and was used to generate LVS P_*FTL_0361*_-PRE-lacZ and LVS ∆*pigR* P_*FTL_0361*_-PRE-lacZ. The pMO7 plasmid contains DNA specifying the FTL_0361 promoter containing the -10 mutant and was used to generate LVS P_*FTL_0361*_-10M-lacZ and LVS ∆*pigR* P_*FTL_0361*_-10M-lacZ.

### Strain construction

Electroporation of integration plasmids into LVS was performed as described [49]. Cells in which a single homologous recombination event had occurred between the integration vector and the chromosome were selected on cysteine heart agar with 1% hemoglobin and either 5 μg/ml (for VSV-G tagging integration vectors) or 10 μg/ml kanamycin (for *lacZ* reporter integration vectors). Strains containing the correct integration were confirmed by colony PCR, by Western blotting and/or Southern blotting. Strain LVS βʹ-V, which synthesizes the βʹ subunit of RNAP with a C-terminal VSV-G tag, was generated by electroporation of plasmid pKL02 into LVS. Strain LVS σ^70^-V, which synthesizes the σ^70^ protein with a C-terminal VSV-G tag, was generated by electroporation of plasmid pKL05 into LVS. Strain LVS σ^32^-V, which synthesizes the σ^32^ protein with a C-terminal VSV-G tag, was generated by electroporation of plasmid pKL04 into LVS. Strain LVS SspA-V, which synthesizes the SspA protein with a C-terminal VSV-G tag, was generated by electroporation of plasmid pKL13 into LVS. Strain LVS PigR-V, which synthesizes the PigR protein with a C-terminal VSV-G tag, was generated by electroporation of plasmid pKL08 into LVS. Strain LVS HipB-V, which synthesizes the HipB protein with a C-terminal VSV-G tag, was generated by electroporation of plasmid pCS05 into LVS.

Strains containing the *iglA*-*lacZ* transcriptional fusion and derivatives were integrated at the FTL_0111 *iglA* locus, as determined by Southern blotting; the probe was synthesized using the PCR DIG Probe Synthesis Kit (Roche), hybridized to digested chromosomal DNA that had been transferred to a positively charged nylon membrane, and detected using CDP-Star (Roche). Strains P_*iglA*_-lacZ and LVS ∆*pigR* P_*iglA*_-lacZ, which contain *lacZ* under the control of the wild-type *iglA* promoter, were generated by electroporation of pMO1 into LVS and LVS ∆*pigR*, respectively. Strains LVS P_*iglA*_-M1-lacZ and LVS ∆*pigR* P_*iglA*_-M1-lacZ, which contain *lacZ* under the control of the *iglA* promoter containing the two mutations in the PRE (PRE mutant 1), were generated by electroporation of pMO2 into LVS and LVS ∆*pigR*, respectively. Strain LVS P_*iglA*_-M2-lacZ, which contain *lacZ* under the control of the *iglA* promoter containing three mutations in the PRE (PRE mutant 2), was generated by electroporation of pMO3 into LVS. Strains LVS P_*iglA*_-10M-lacZ and LVS ∆*pigR* P_*iglA*_-10M-lacZ, which contain *lacZ* under the control of the *iglA* promoter containing two mutations in the -10 element, were generated by electroporation of pMO4 into LVS and LVS ∆*pigR*, respectively. Strains LVS P_*FTL_0361*_-lacZ and LVS ∆*pigR* P_*FTL_0361*_-lacZ, which contain *lacZ* under the control of the FTL_0361 promoter, were generated by electroporation of pMO5 into LVS and LVS ∆*pigR*, respectively. Strains LVS P_*FTL_0361*_-PRE-lacZ and LVS ∆*pigR* P_*FTL_0361*_-PRE-lacZ, which contain *lacZ* under the control of the FTL_0361 promoter containing the PRE, were generated by electroporation of pMO6 into LVS and LVS ∆*pigR*, respectively. Strains LVS P_*FTL_0361*_-10M-lacZ and LVS ∆*pigR* P_*FTL_0361*_-10M-lacZ, which contain *lacZ* under the control of the FTL_0361 promoter containing mutations in the -10 element, were generated by electroporation of pMO7 into LVS and LVS ∆*pigR*, respectively.

### Ectopic *pigR* expression

Plasmids pF and pF-PigR-V have been described previously [17] and were used as a negative control vector and to drive ectopic expression of PigR with a C-terminal VSV-G tag, respectively. The pF-PigR-V plasmid contains DNA encoding the PigR protein fused to the VSV-G epitope, which is driven from the *groES* promoter; the pF plasmid does not contain the *pigR* gene or DNA encoding the VSV-G epitope tag. Plasmid pF2-PigR-V synthesizes PigR-V under the control of a weakened *groES* promoter lacking its putative UP-element and was made by replacing *sspA* in the plasmid pF2-SspA [15] with DNA encoding PigR-V. These plasmids were electroporated into either cells of the previously described LVS ∆*pigR* mutant strain [17], or cells of a LVS ∆*pigR* ∆*mglA* mutant strain; LVS ∆*pigR* ∆*mglA* was created by using the pEX2-∆*mglA* vector [15] in the ∆*pigR* background, by allelic exchange and confirmed by Southern blotting.

### ChIP-Seq

ChIP-Seq was performed with cells of the following strains: LVS βʹ-V; LVS σ^70^-V; LVS σ^32^-V; LVS PigR-V; LVS SspA-V; LVS HipB-V; LVS (as a mock control); LVS containing plasmid pF (as a mock control); LVS ∆*pigR* containing plasmid pF2-PigR-V; and LVS ∆*pigR* ∆*mglA* containing plasmid pF-PigR-V. In order to perform ChIP-Seq on MglA, we used cells of the LVS strain synthesizing MglA with a C-terminal TAP tag (LVS MglA-TAP) at native levels, which has been described previously [15]. Cells were grown at 37°C in 100 mL of supplemented MH to mid-log (OD_600_ 0.3–0.4), and when indicated, rifampicin (Sigma) was added to a final concentration of 50 μg/mL for 30 minutes before crosslinking. Cells were incubated in a final concentration of 1% formaldeyhyde (Sigma) for 30 minutes, after which glycine (Sigma) was added to a final concentration of 250 mM. ChIP was performed in biological triplicate (excepting β′ + rifampicin, the LVS pF empty vector control, and LVS ∆*pigR* ∆*mglA* pF-PigR-V, which were performed in duplicate, and σ^70^, which was performed in quadruplicate) with either 40 mL or 80 mL of culture using anti-VSV-G agarose beads (Sigma) for cells synthesizing VSV-G tagged transcription factors or IgG Sepharose beads (GE Healthcare) for cells synthesizing TAP-tagged MglA essentially as described previously [50], except that a water bath sonicator (Biorupter, Diagenode) was used to lyse cells and shear chromosomal DNA to 200 to 500 bp. Immunoprecipitated DNA was quantified using the Quant-iT PicoGreen dsDNA Assay Kit (Invitrogen). The same protocol was performed with the untagged LVS strain as a mock immunoprecipitation (mock IP) control.

Illumina libraries were constructed with approximately 2 to 160 ng immunoprecipitated DNA using either the TruSeq DNA Sample Prep Kit (Illumina) or the NEBNext Ultra DNA Library Prep Kit for Illumina (NEB), generally following the supplied protocols. In using the TruSeq DNA Sample Prep Kit, adapters were diluted 1:10 before ligation and libraries were gel-purified after 11 cycles of amplification. When using the NEBNext Ultra DNA Library Prep Kit, adapters were diluted 1:10 before ligation and libraries were size-selected using Agencourt AMPure XP beads prior to 12 cycles of amplification. Libraries were quantified using the Quant-iT PicoGreen dsDNA Assay Kit (Life Technologies) and sequenced by Elim Biopharmaceuticals, Inc. (Hayward, CA), using an Illumina Genome Analyzer Ilx generating 36 bp single-end reads or an Illumina HiSeq 2500 generating 50 bp single-end reads. Sequencing reads have been submitted to the NCBI Sequence Read Archive (SRA, http://www.ncbi.nlm.nih.gov/Traces/sra) with the accession number SRP055716.

### ChIP-Seq data analysis

For each strain, the reads were mapped to the *F*. *tularensis* subsp. *holarctica* LVS genome (NCBI locus AM233362) and the sequence of the integrated plasmid, if applicable, using bowtie2-2.0.6 [51]. Regions of enrichment were called using QuEST, version 2.42 [52]. The three mock IP biological replicates, consisting of approximately 30.6 million reads, were merged and used as a background control for each biological replicate. The two pF empty vector control IP biological replicates, consisting of approximately 42.7 million reads, were merged and used as a background control for the ectopic PigR-V experiments. Peaks in each biological replicate are regions that fit the following criteria: they are 1.5-fold enriched for reads over background, with a positive peak shift and strand correlation, and a q-value of less than 0.01. Peaks for each immunoprecipitated protein were defined as the maximal region identified in at least two biological replicates. Promoter regions are defined as the maximal regions of enrichment of βʹ plus rif, σ^70^ or σ^32^. Tracks were visualized using the Integrative Genomics Viewer (IGV), version 2.3 [53]. Peak analyses were carried out using Perl scripts, samtools, version 0.1.17 [54], and BEDtools, version 2.17.0 [55].

### Immunoblots

Cell lysates were separated by SDS-PAGE on 4–12% or 12% Bis-Tris NuPAGE gels in MES or MOPS running buffer (Life Technologies). Either the iBlot dry blotting system or the XCell II Blot Module (Life Technologies) was used to transfer proteins to either PVDF or nitrocellulose. Membranes were blocked with SuperBlock Blocking Buffer (Pierce) with 0.25% Surfact-Amps 20 (Pierce) for 1 hour to overnight. Membranes were then probed with polyclonal anti-VSV-G (diluted 1:1,500; Sigma) or anti-GroEL (diluted 1:160,000; provided by Karsten Hazlett, Albany Medical College, Albany, New York, United States) for one hour, washed (10 minutes incubations in TBST plus 0.25% Surfact-Amps 20, 4 times) and re-blocked for 1 hour. After membranes were incubated with polyclonal goat anti-rabbit (diluted 1:10,000; Pierce) and washed, proteins were detected using SuperSignal West Pico Chemiluminescent Substrate (Life Technologies).

### Transcriptomic analysis

Cells of the LVS wild-type strain and cells of the LVS *∆pigR* mutant strain (described in 17) were grown to mid-log in biological triplicate. 1 mL of each sample was pelleted (20,000 rcf for 5 minutes), resuspended in 500 μL Qiagen buffer RLT, frozen on dry ice, and stored at -80°C. Equal amounts of lysate, normalized to OD_600_, in 4 μL Qiagen buffer RLT and 1 μL water were submitted to the Epithelial Cell Biology Core Facility (Boston Children’s Hospital) for processing using the Nanostring nCounter Prep Station and Digital Analyzer according to the manufacturer’s instructions. For each replicate, total transcript counts were normalized using internal controls with background subtraction, as per manufacturer’s instructions. Transcript abundance was determined by averaging biological triplicates. Criteria indicating a significant change in gene expression are a 2-fold change in transcript abundance and p-value < 0.05 in a two-tailed Student’s t-test.

### Identifying the PRE

Genes with significant changes in expression in cells lacking PigR in comparison to wild-type cells (greater than 3-fold by microarray [17] or 2-fold by Nanostring) were examined for promoter regions with detectable PigR, identifying 11 genes (FTL_0026, *iglA*, *pdpA*, FTL_0131, FTL_0207, FTL_0449, FTL_0491, FTL_1218, FTL_1219, FTL_1678, FTL_1790). The 400 bp region surrounding the maximal PigR binding site, upstream from 11 genes, excluding coding regions, was searched for a common motif using MEME, version 4.9.1 [28]. We have named the second result, which was present in all 11 promoters and consisted of the consensus sequence TGCTGTA, the PigR response element (PRE).

### Identifying transcription start sites using RNA-Seq

LVS cells were grown in aerated liquid culture at 37°C in supplemented MH to mid-log (OD_600_ 0.3–0.4), and RNA was isolated from 10mL of cells in triplicate as described previously [18]. RNA-Seq was used to identify transcription start sites. In particular, from each sample we prepared three cDNA libraries derived from the 5’ ends of RNAs. The first library was generated from RNAs carrying a 5’ triphosphate (prepared as described in [56]), the second library was generated from RNAs carrying a 5’ monophosphate (prepared as described in [56]), while the third library was generated from RNAs carrying either a 5’ triphosphate or a 5’ monophosphate. The third library was prepared by omitting a single step (treatment with Terminator 5’ exonuclease) from the procedure used to generate RNAs carrying a 5’ triphosphate. To identify high quality transcription start sites we first identified 3,120 genomic loci where 50 or more sequencing reads aligned in one of three libraries generated from RNAs carrying a 5’ triphosphate. Of these 3,120 loci, we identified 452 sites that were significantly enriched in a comparison of libraries generated from RNAs carrying a 5’ triphosphate with libraries generated from RNAs carrying a 5’ monophosphate and significantly enriched in a comparison of libraries generated from RNAs carrying a 5’ triphosphate or a 5’ monophosphate with libraries generated from RNAs carrying a 5’ monophosphate. These 452 sites were further filtered to remove those associated with rRNAs, tRNAs, or repeated sequences annotated as transposases. Among the remaining sites, we identified 110 high quality start sites as those found within 1 kb of a translational start site for an annotated ORF and located within a promoter region defined by ChIP-Seq (S8 Table).

### Primer extension analysis

Primer extension was used essentially as described previously [57] to determine the putative transcription start sites for *pdpA* and FTL_1219.

### Identifying the location of the PRE

For each transcription start site identified by RNA-Seq, putative -10 and -35 elements were predicted based on homology to the *E*. *coli* consensus sequence. For each promoter, 11 bp of sequence, 5 bp upstream from the -35 element and extending upstream, was submitted to FIMO [58] to search for the PRE. Only three promoters were found to contain the PRE with a p-value <0.001, all of which are known to be PigR-regulated. None of the remaining promoters are known to be PigR-regulated and none of them contained the PRE (p<0.001).

### β-galactosidase assays

Cells were grown to mid-log phase, and β-galactosidase activity was assessed essentially as described previously [17]. Assays were performed at least twice in triplicate on separate occasions. Representative data sets are shown. Values are averages based on one experiment.

## Supporting Information

S1 Fig HipB is specifically enriched upstream of its own gene. A representative track illustrating the density of normalized mapped sequencing reads is depicted on the Y-axis after ChIP-Seq of each epitope-tagged factor: β′ (green), β′+ rif (purple), σ^70^ (orange), σ^32^ (cyan), MglA (brown), SspA (light pink), PigR (blue), and HipB (dark pink). The region of the chromosome illustrated includes the *hipAB* operon (*hipA* is FTL_1125 and *hipB* is FTL_1126).
(EPS)

S2 Fig The abundance of ectopically expressed PigR-V is similar in cells lacking PigR and cells lacking both PigR and MglA. Abundance of ectopically expressed PigR-V in biological replicate samples used for ChIP-Seq shown in Fig 3B and 3C as analyzed by western blot. (*Upper*) Western blot probed with antibody against VSV-G tag. (*Lower*) Western blot probed with antibody against GroEL serves as a loading control. Wild-type LVS cells containing the empty control vector pF (lanes 1–3); cells of LVS ∆*pigR* containing plasmid pF2-PigR-V (lanes 4–6); cells of LVS ∆*pigR* ∆*mglA* containing plasmid pF-PigR-V (lanes 7–9).
(EPS)

S1 Table Locations of significant enrichment of β′ in cells treated with rifampicin (β′+rif) as determined by ChIP-Seq. *Enrichment is determined with respect to the mock control. **Genes are considered to be associated with a ChIP-Seq peak if the translation start site is within 100 bp upstream to 500 bp downstream of the position of maximal enrichment.
(XLSX)

S2 Table Locations of significant enrichment of σ^70^ as determined by ChIP-Seq. *Enrichment is determined with respect to the mock control. **Genes are considered to be associated with a ChIP-Seq peak if the translation start site is within 100 bp upstream to 500 bp downstream of the position of maximal enrichment.
(XLSX)

S3 Table Locations of significant enrichment of σ^32^ as determined by ChIP-Seq. *Enrichment is determined with respect to the mock control. **Genes are considered to be associated with a ChIP-Seq peak if the translation start site is within 100 bp upstream to 500 bp downstream of the position of maximal enrichment.
(XLSX)

S4 Table Locations of significant enrichment of HipB as determined by ChIP-Seq. *Enrichment is determined with respect to the mock control. **Genes are considered to be associated with a ChIP-Seq peak if the translation start site is within 100 bp upstream to 500 bp downstream of the position of maximal enrichment.
(XLSX)

S5 Table Promoter regions in *F*. *tularensis* LVS as defined by ChIP-Seq of β′+rif, σ^70^, and σ^32^. There are 581 promoter regions reported (P1-P581), which were identified by ChIP-Seq of one or more factors (β′+rif, σ^70^, and σ^32^). Promoters are listed multiple times if more than one transcription factor and ChIP-Seq peak was used to define the promoter. The nucleotide location on the genome is reported for each promoter location (“Start” and “End”). The factors used to define each promoter are indicated (“Factor used to define promoter”): βʹ+rif (“Betaprime+rif”), σ^70^ (“Sigma70”), or σ^32^ (“Sigma32”). *Each factor listed refers to a particular ChIP-Seq peak for the given factor within the defined promoter region. Factors may be listed more than once for a particular promoter if multiple ChIP-Seq peaks are included in one promoter region. **Nucleotide position of maximal enrichment of ChIP-Seq peak for indicated factor. ***Genes are considered to be associated if the translation start site is within 100 bp upstream to 500 bp downstream of the position of maximal enrichment.
(XLSX)

S6 Table Locations of significant enrichment of PigR as determined by ChIP-Seq. *Enrichment is determined with respect to the mock control. **Genes are considered to be associated with a ChIP-Seq peak if the translation start site is within 100 bp upstream to 500 bp downstream of the position of maximal enrichment.
(XLSX)

S7 Table Fold change of transcripts of select genes in cells lacking PigR compared to wild-type, as determined by Nanostring. This table presents an analysis of the effects of *pigR* on the expression of 100 genes in LVS using Nanostring. The limited gene set used in this analysis includes a representative sample of 19 genes shown previously to be positively regulated by MglA/SspA and PigR [15–17], but also includes many other genes that served as negative controls. Of the 19 genes in this set that were found previously to be positively regulated by MglA, SspA, and PigR, 16 were found here to be regulated by PigR. The reported fold change is highlighted if larger than 2-fold and the p-value is < 0.05. *indicates statistically significant changes in gene expression (p <0.05) as determined by a two-tailed homoscedastic t-test.
(XLSX)

S8 Table High confidence transcription start sites in *F*. *tularensis* LVS as determined by RNA-Seq. *Transcription start site is indicated in uppercase. **Identified PRE motifs are in bold. ***Predicated extended -10 elements are in bold.
(XLSX)
